# Supplementary material for: Colloidal Quasi‐2D Methylammonium Lead Bromide Perovskite Nanostructures with Tunable Shape and High Chemical Stability
Source: Small. 2024 Sep 17;20(48):2405758. doi: 10.1002/smll.202405758 (PMC11600696; doi:10.1002/smll.202405758)
Supplement: Supplementary file 1 — Supporting Information [file SMLL-20-2405758-s001.pdf]

NANO · MICRO
small

Supporting Information

for *Small*, DOI 10.1002/smll.202405758

Colloidal Quasi-2D Methylammonium Lead Bromide Perovskite Nanostructures with Tunable Shape and High Chemical Stability

*Eugen Klein, Rostyslav Lesyuk and Christian Klinke**

Colloidal quasi-2D Methylammonium Lead Bromide Perovskite Nanostructures with Tunable Shape and High Chemical Stability

Eugen Klein,[1] Rostyslav Lesyuk,[1,2] Christian Klinke[1,3,4]*

[1] *Institute of Physics, University of Rostock, Albert-Einstein-Straße 23, 18059 Rostock, Germany*
[2] *Pidstryhach Institute for applied problems of mechanics and mathematics of NAS of Ukraine, Naukowa str. 3b, 79060 Lviv, Ukraine*
[3] *Department "Life, Light & Matter", University of Rostock, Albert-Einstein-Straße 25, 18059 Rostock, Germany*
[4] *Department of Chemistry, Swansea University – Singleton Park, Swansea SA2 8PP, United Kingdom*
* Corresponding author: christian.klinke@uni-rostock.de

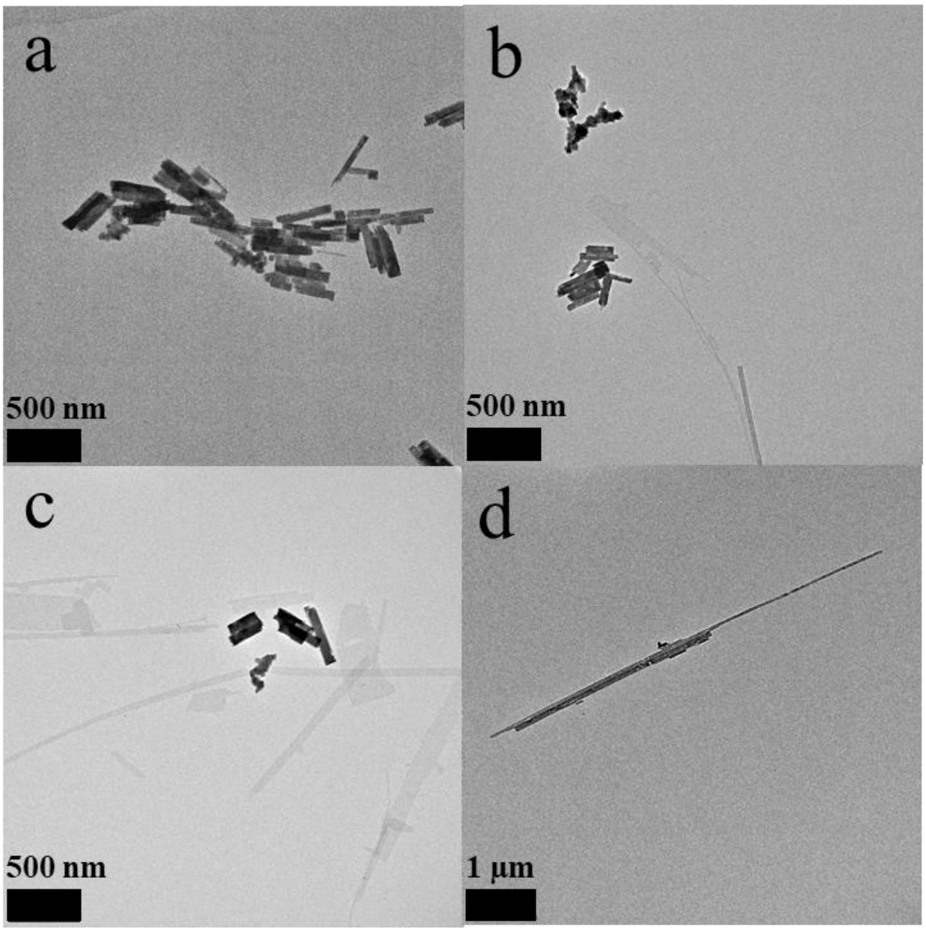

Figure S1. Synthesis progress with time. (a) Right after the injection of the methylammonium bromide precursor, (b) 10 s, (c) 20 s and (d) end product.

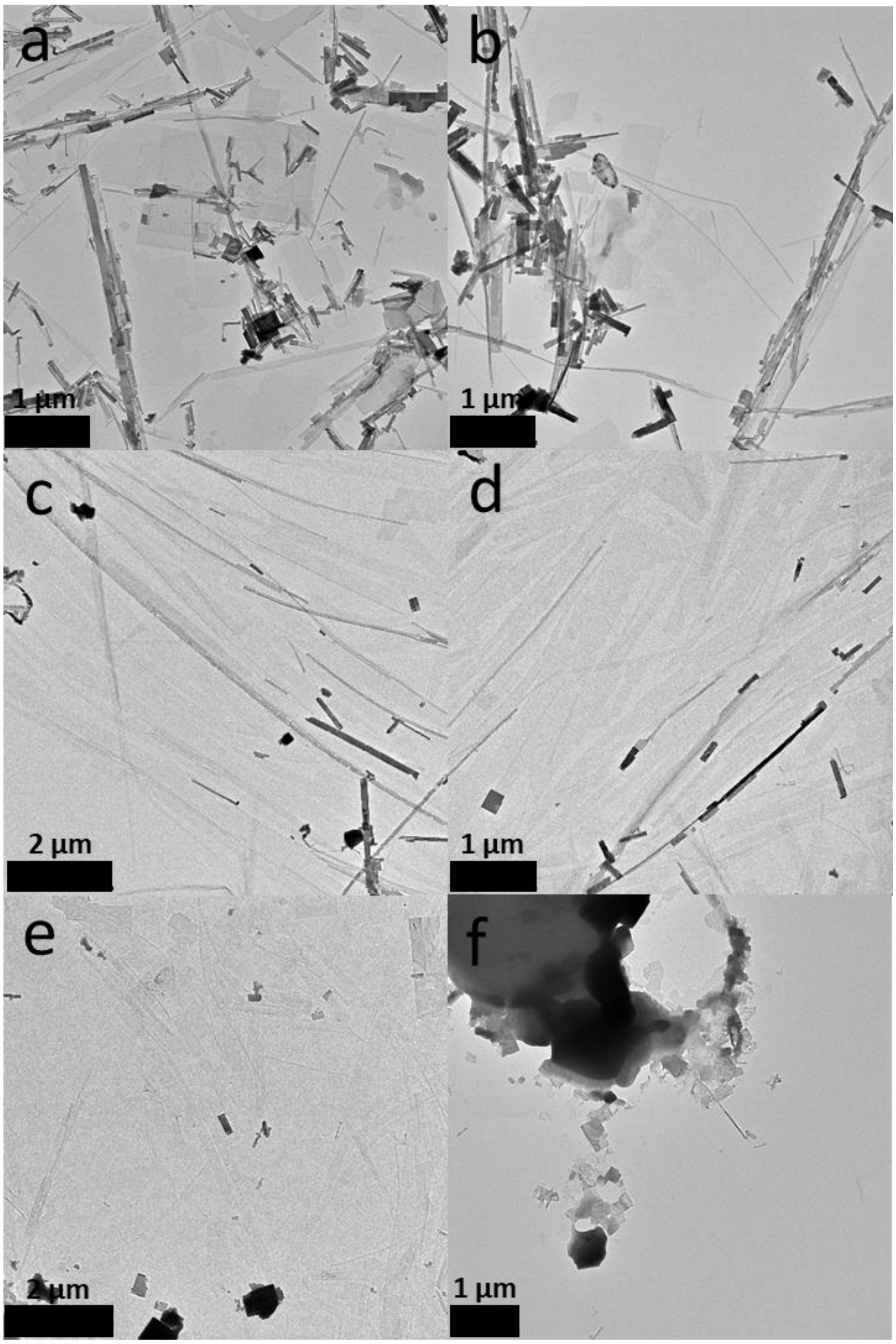

Figure S2. BF-TEM images of MAPbBr nanoparticles prepared with a different amount of DPE, (a) 2 mL, (b) 4 mL (c) 6 mL, (d) 8 mL, (e) 12 mL, (f) 20 mL.

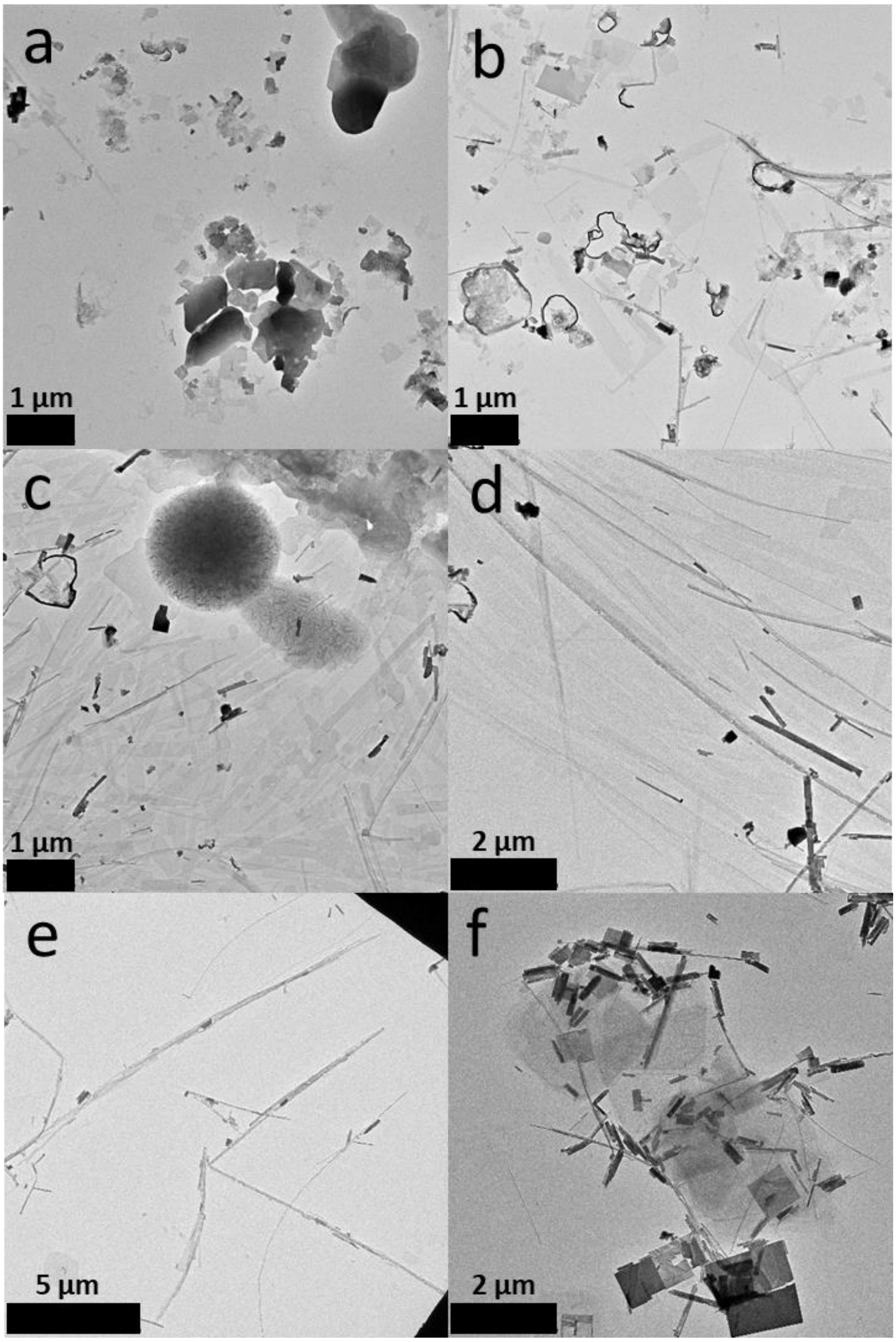

Figure S3. BF-TEM images of MAPbBr nanoparticles prepared at different temperatures, (a) 100 °C, (b) 110 °C (c) 120 °C, (d) 130 °C, (e) 140 °C, (f) 150 °C.

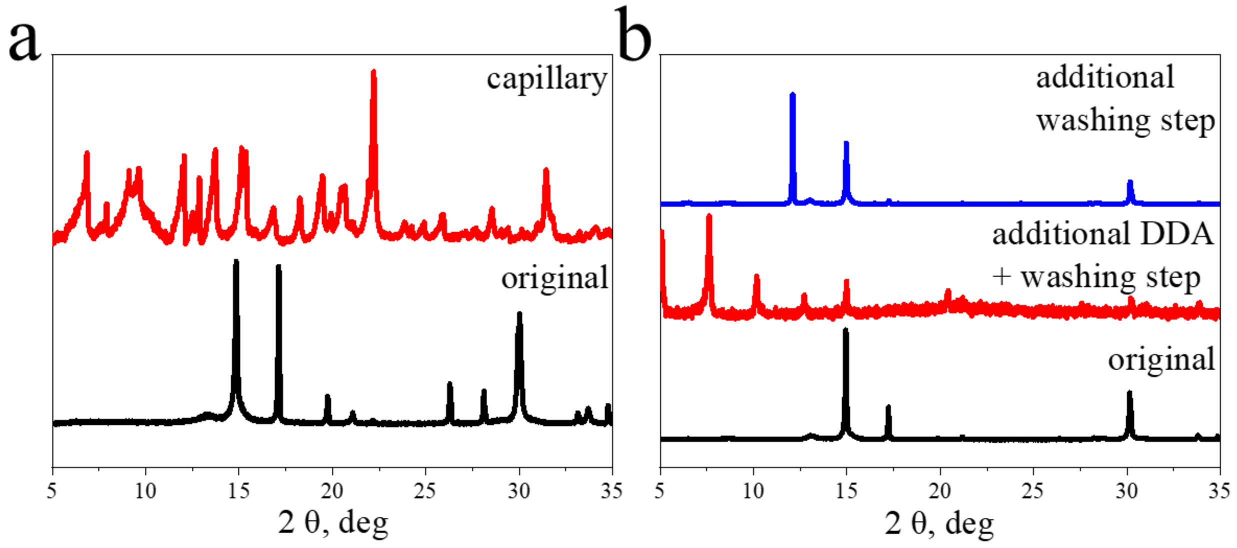

Figure S4. (a) XRD patterns of MAPbBr nanostripes measured on a conventional wafer (original) and in a capillary. (b) XRD patterns of the same sample untreated (original), with one additional washing step and with additional DDA.

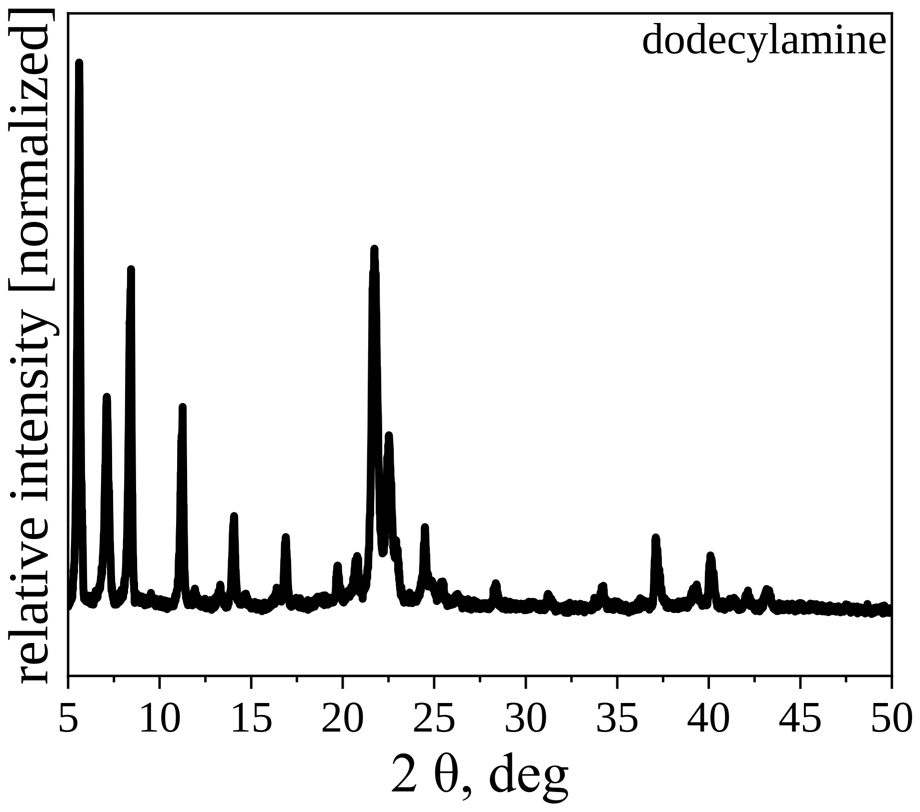

Figure S5. XRD pattern of polycrystalline dodecylamine measured on a conventional wafer.

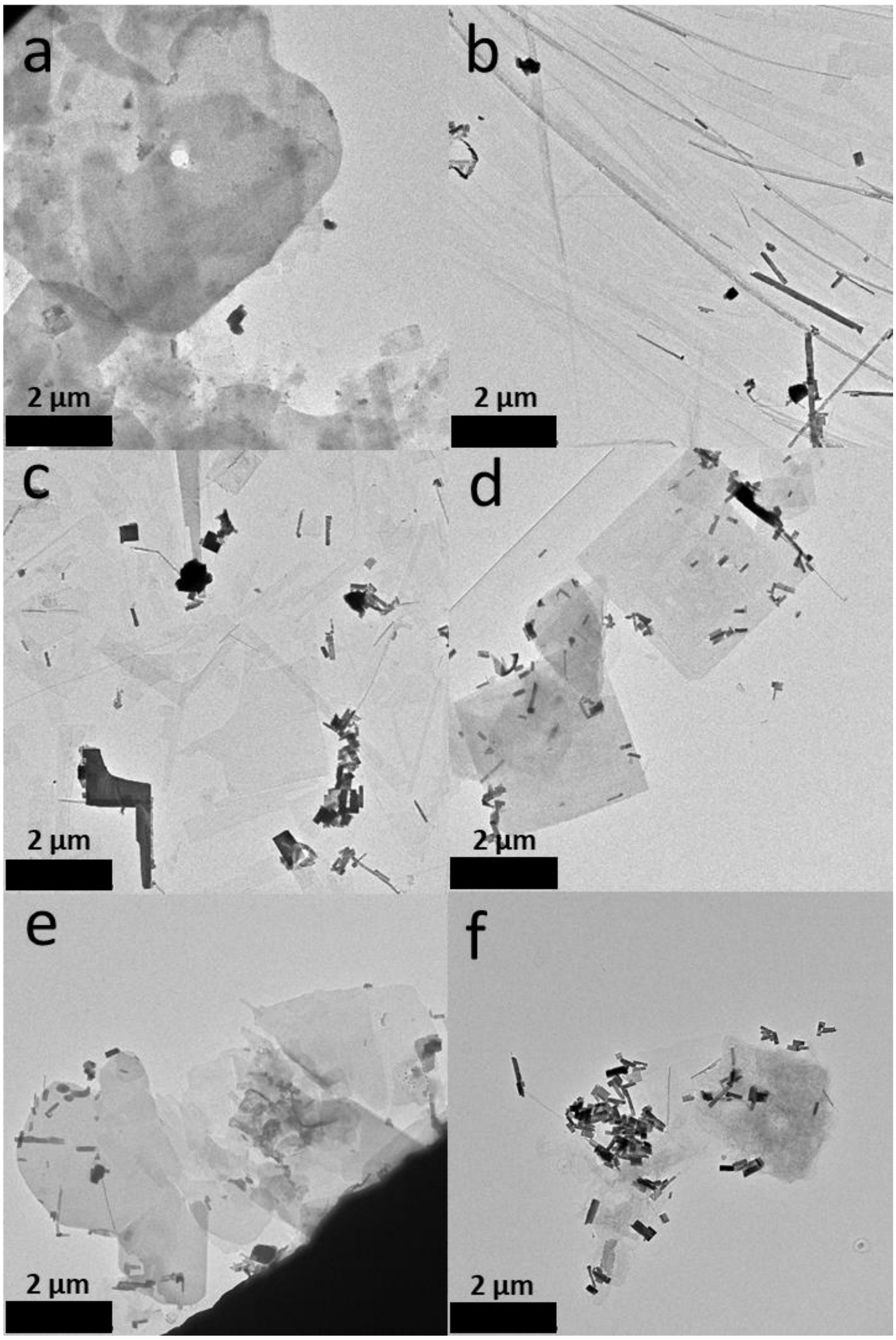

Figure S6. BF-TEM images of MAPbBr nanoparticles prepared with different amines. The molar mass was fix for all of these experiments at 0.055 mmol. (a) octylamine, (b) dodecylamine, (c) tetradecylamine, (d) hexadecylamine, (e) octadecylamine, (f) oleylamine.

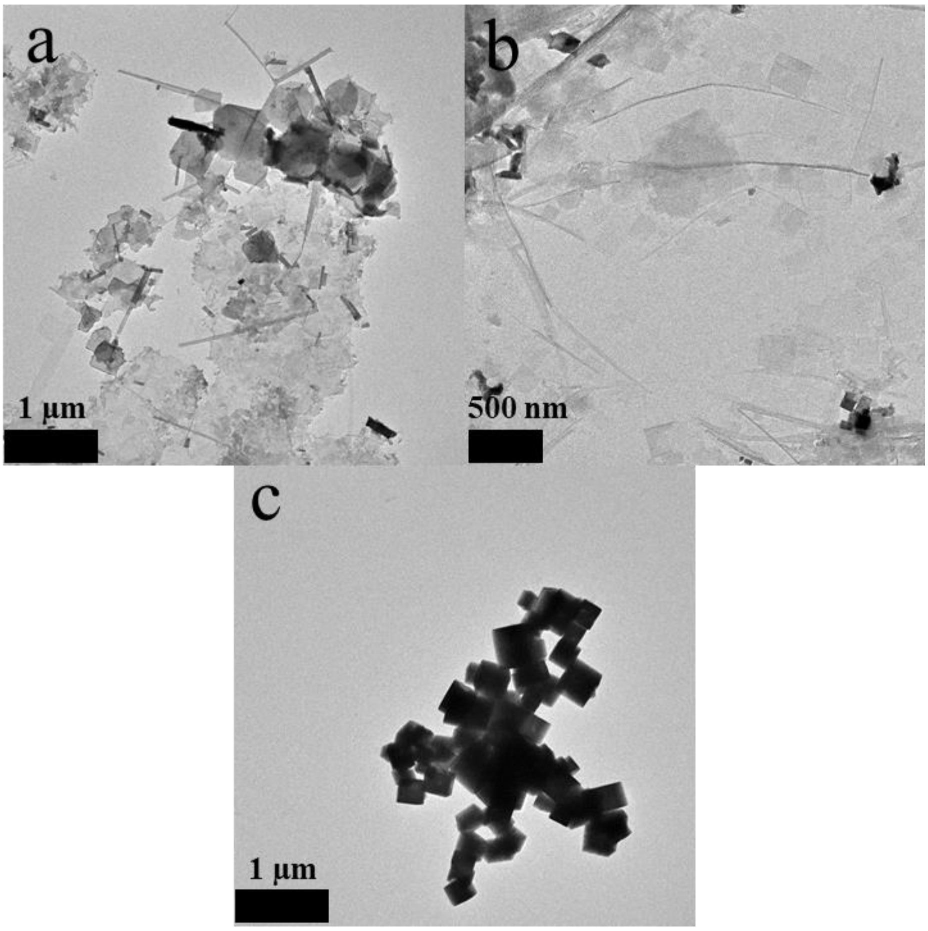

Figure S7. Tests regarding the synthesis reactivity. (a) Standard nanostripe synthesis without TOP stopped immediately after the injection of MAB. (b) Standard nanostripe synthesis without TOP but with 0.4 mL of DDA. (c) Standard nanostripe synthesis without amine but with 0.5 mL of TOP.

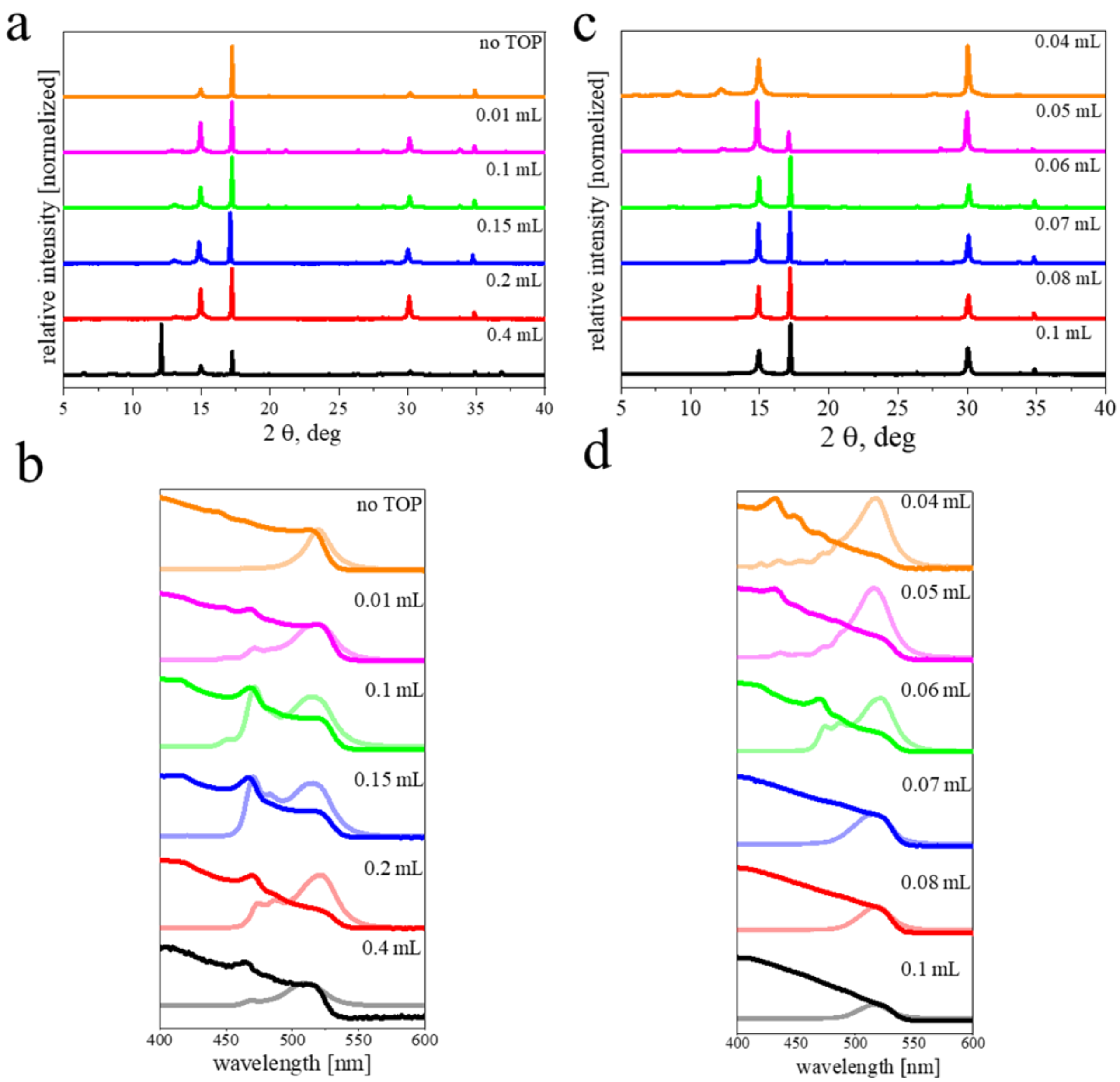

Figure S8. XRD, UV-Vis and PL data for a different amount of the co-ligand TOP (a, b) and the second precursor methylammonium iodide (c, d).

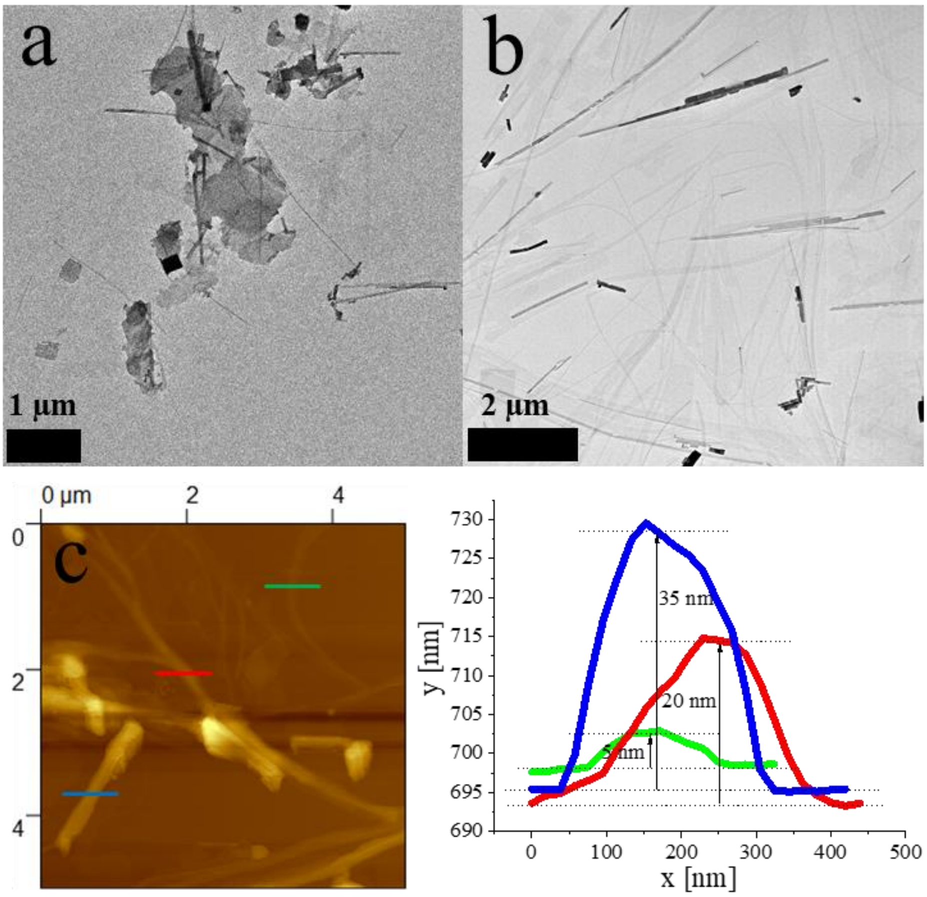

Figure S9. (a, b) Nanostripe syntheses with 0.04 mL and 0.08 mL of MAB-precursor stopped with a water bath cooling immediately after the injection of the second precursor. (c) AFM image and height profile of the product from the 0.08 mL synthesis.

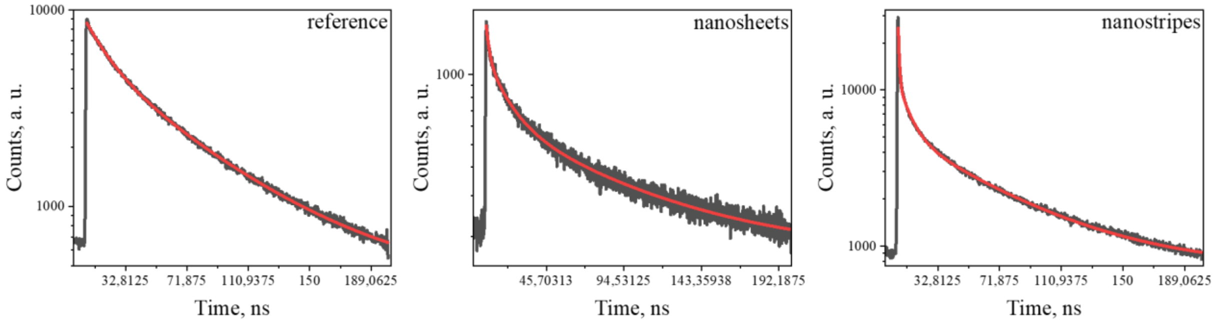

Figure S10. Photoluminescence lifetime of nanosheet reference, nanosheets and nanostripes (black lines) with corresponding fits (red lines) .

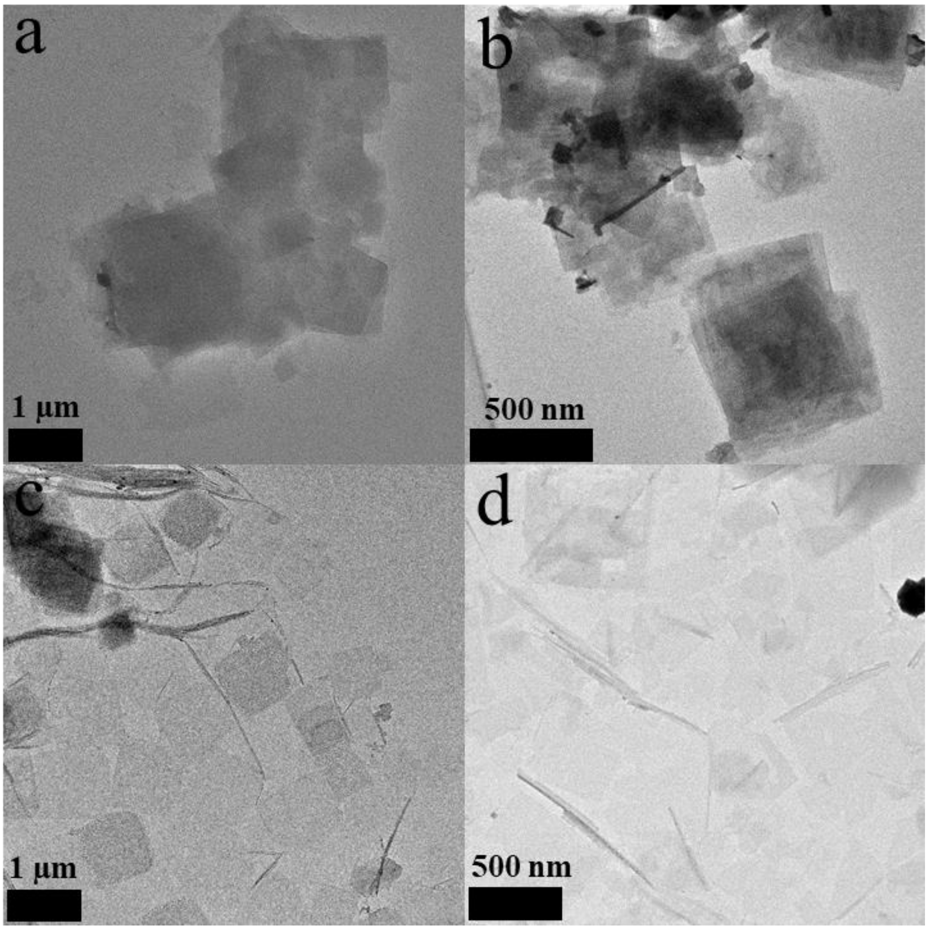

Figure S11. BF-TEM images of MAPbBr nanoparticles prepared with different synthesis protocols, (a, b) nanosheets one day after the synthesis and two months later, (c, d) nanosheets with a minor fraction of nanostripes prepared in a similar way like the standard nanostripes one day after the synthesis and two months later.

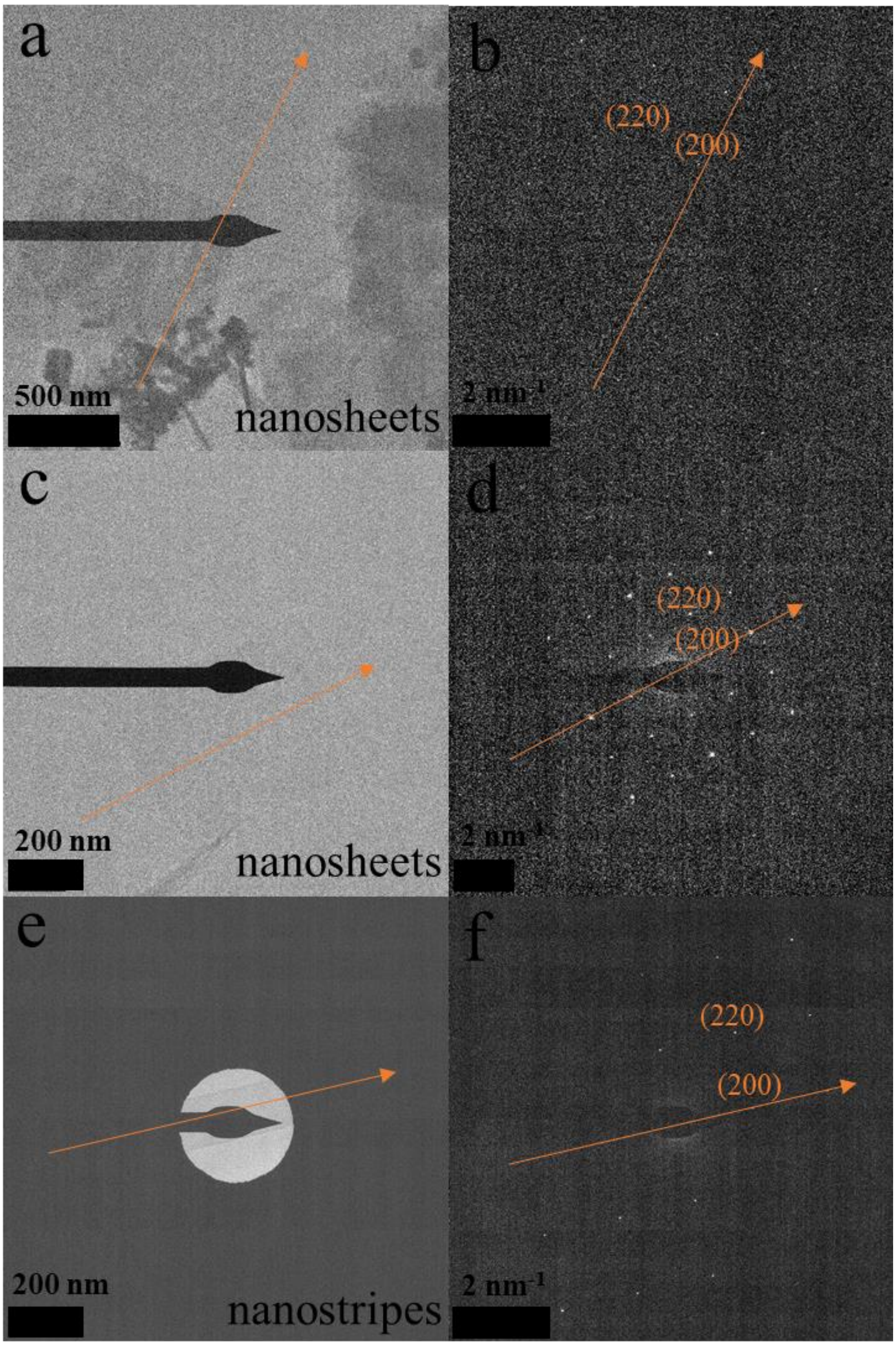

Figure S12. TEM images of MAPbBr nanoparticles (a, c, e) with corresponding SAED pattern (b, d, f), indicating the <100> zone axis and edges in <110> direction.

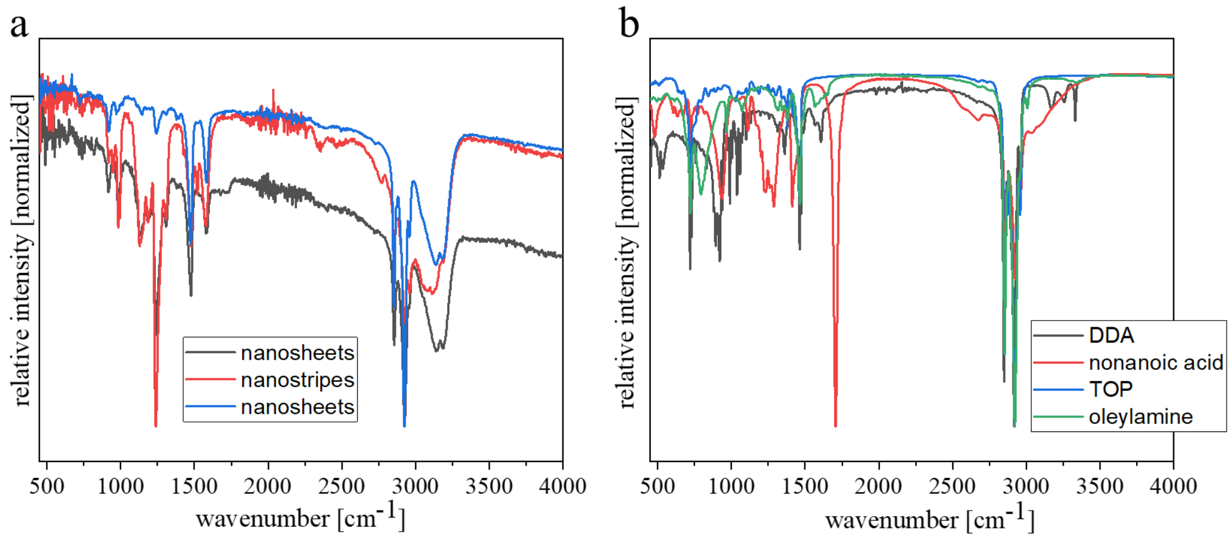

Figure S13. FTIR spectra of MAPbBr nanoparticles (a) and the ligands used in the syntheses (b). Nanosheets prepared with a different synthesis protocol (black), nanosheets prepared with a higher amount of DDA (blue), nanostripes (red).
